# Telemedicine for Initiation of Alcohol Use Disorder Medications

**DOI:** 10.1001/jamanetworkopen.2024.31594

**Published:** 2024-09-04

**Authors:** Haiden A. Huskamp, Lori Uscher-Pines, Pushpa Raja, Sharon-Lise T. Normand, Ateev Mehrotra, Alisa B. Busch

**Affiliations:** 1 Department of Health Care Policy, Harvard Medical School, Boston, Massachusetts; 2 RAND Corporation, Arlington, Virginia; 3 Veterans Affairs Greater Los Angeles Healthcare System, Los Angeles, California; 4 McLean Hospital, Belmont, Massachusetts

## Abstract

This case-control study examines the initiation of treatment with medications for alcohol use disorder (MAUD) among US adults and compares the characteristics of adults who initiate MAUD treatment via telemedicine vs in-person care.

## Introduction

Alcohol use disorder (AUD) is the most prevalent substance use disorder (SUD) in the US, affecting 29.5 million people, and deaths from excessive alcohol use are increasing.^1,2^ While medications for alcohol use disorder (MAUD) are efficacious^3^ and can be prescribed in primary care or specialty behavioral health settings, only 2.2% of individuals with AUD in 2022 received MAUD in the past year.^1^ Barriers to MAUD initiation include clinician shortages, cost, stigma and/or privacy concerns, and lack of transportation^4^; however, telemedicine could reduce some of these barriers.^5^ We examined telemedicine initiation of MAUD and compared the characteristics of US adults who initiate via telemedicine vs in-person care.

## Methods

This case-control study was approved by the Harvard University Faculty of Medicine Institutional Review Board, who waived the need for informed consent owing to the use of deidentified claims data. We followed the STROBE reporting guideline.

We used claims data obtained from Optum Labs Data Warehouse for adults aged 18 to 64 years in all 50 states who had commercial or Medicare Advantage insurance from January 2019 to September 2023. Initiation of MAUD was identified using the first prescription fill for naltrexone, acamprosate, disulfiram, or topiramate during the period with no fills in the previous 90 days (hereinafter, the index fill date) and categorized as telemedicine if all linked outpatient visits 7 days before or 3 days after the index fill date were virtual (eMethods in Supplement 1). We required continuous enrollment in medical, behavioral health, and pharmacy benefits in the index fill month and prior 6 months to identify baseline characteristics. We excluded people who did not have an AUD diagnosis in the 180 days before and including the index fill date.

We examined the monthly proportion of initiations via telemedicine. Because telemedicine was uncommon before the COVID-19 pandemic, we focused on initiations that occurred from April 2020 to September 2023, using logistic regression (via SAS, version 9.4 [SAS Institute Inc]) to identify patient characteristics associated with telemedicine (vs in-person) initiation. The model included age; sex; US region; rural residence; household income quartile in county of residence; claims with a non-AUD SUD, mental health, or acute or chronic alcohol-related medical condition diagnosis; and clinician specialty (eMethods in Supplement 1). We used 2-sided testing with a significance threshold of *P* < .05.

## Results

There were 19 121 MAUD initiations from January 2019 to September 2023 (patient characteristics are provided in the Table). Of these initiations, 68.5% were for oral naltrexone, 10.8% were for acamprosate, 7.8% were for disulfiram, 10.6% were for topiramate, and 2.3% were for injectable naltrexone; 58.6% were conducted by PCPs, 25.5% by psychiatrists, and 15.9% by other prescribers. Telemedicine was used in 13.9% of all initiations, reaching a peak of 26.4% in May 2020 and plateauing at 16.6% by September 2023 (Figure). Telemedicine initiations differed across drugs (naltrexone, 14.6%; topiramate, 11.8%; disulfiram, 12.8%; and acamprosate, 13.5%).

**Table.** Patient Characteristics and Odds of Initiation of Medications for AUD via Telemedicine vs In-Person Care, April 2020 to September 2023

Characteristic	No. (%) of patients (N = 13 961)	AOR (95% CI)	*P* value
Age, y			
18-25	1119 (8.0)	1.16 (0.96-1.39)	.12
26-40	4305 (30.8)	1.72 (1.54-1.93)	<.001
41-50	3774 (27.0)	1.41 (1.25-1.59)	<.001
51-64	4763 (34.1)	1 [Reference]	NA
Sex			
Female	5848 (41.9)	1 [Reference]	NA
Male	8113 (58.1)	0.80 (0.73-0.87)	<.001
US region			
Midwest	4217 (30.2)	0.75 (0.67-0.84)	<.001
West	3097 (22.2)	1.10 (0.98-1.24)	.11
Northeast	4975 (35.6)	1.27 (1.10-1.47)	.001
South	1672 (12.0)	1 [Reference]	NA
Rural residence			
Yes	309 (2.2)	1.1 (0.80-1.52)	.56
No	13 652 (97.8)	1 [Reference]	NA
Specialty of initiating clinician			
Psychiatrist	3555 (25.5)	1.56 (1.41-1.72)	<.001
Primary care physician	8180 (58.6)	1 [Reference]	NA
Other	2226 (15.9)	0.86 (0.75-0.99)	<.001
Non-AUD SUD diagnosis			
Yes	3928 (28.1)	0.86 (0.77-0.95)	.003
No	10 033 (71.9)	1 [Reference]	NA
Mental health diagnosis			
Yes	2943 (21.1)	1.98 (1.73-2.26)	<.001
No	11 018 (78.9)	1 [Reference]	NA
Alcohol-related medical condition diagnosis			
Yes	1391 (10.0)	0.73 (0.62-0.87)	<.001
No	12 570 (90.0)	1 [Reference]	NA
Household income quartile in county of residence			
1st	NA	1 [Reference]	NA
2nd	NA	1.34 (1.17-1.54)	<.001
3rd	NA	1.38 (1.21-1.58)	<.001
4th	NA	1.71 (1.50-1.96)	<.001
Month	NA	0.97 (0.96-0.99)	<.001
Month squared	NA	1.00 (1.00-1.001)	.01

Abbreviations: AOR, adjusted odds ratio; AUD, alcohol use disorder; NA, not applicable; SUD, substance use disorder.

**Figure.**
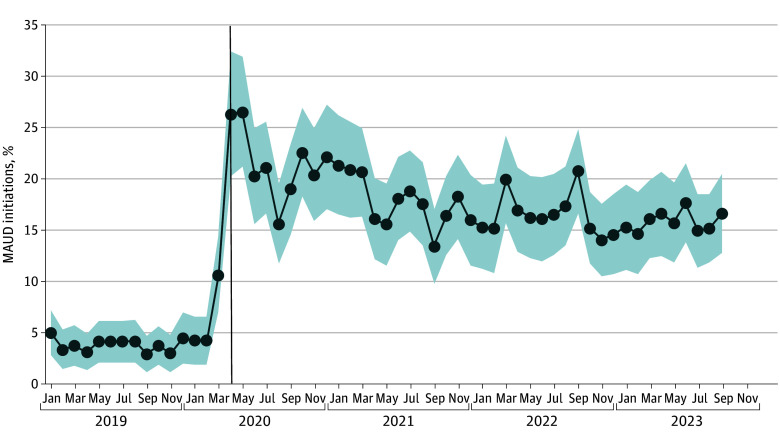
Initiations of Medications for Alcohol Use Disorder (MAUD) Conducted via Telemedicine, January 2019 through September 2023 Due to small sample sizes, data for June 2019, July 2019, and February 2020 were not available. Instead, the value for the most recent available month prior to that month was substituted. The vertical line indicates the declaration of the COVID-19 pandemic. The shading represents the 95% CIs for each month.

Among 13 961 initiations from April 2020 to September 2023, in adjusted analyses, telemedicine was more common among psychiatrists (adjusted OR [AOR] [95% CI], 1.56 [1.41-1.72]) than primary care physicians, enrollees with a mental health diagnosis (AOR, 1.98 [95% CI, 1.73-2.26]), and in higher-income relative to lower-income areas (AOR, 1.71 [95% CI, 1.50-1.96]) (Table). Telemedicine MAUD initiation was lower among men than women (AOR, 0.80 [95% CI, 0.73-0.87]), patients with a non-AUD SUD (AOR, 0.86 [95% CI, 0.77-0.95]), and patients with an alcohol-related medical condition (AOR, 0.73 [95% CI, 0.62-0.87]) (Table).

## Discussion

The findings of this case-control study suggest that while most MAUD initiations occur in person, telemedicine may play an important role in the initiation of these efficacious medications. Greater telemedicine use in higher-income areas supports concerns about how the “digital divide” may decrease access to care.^6^ Study limitations include findings that may not be generalizable to other populations, lack of race and ethnicity information, and an inability to observe initiations for people who pay fully out of pocket for outpatient AUD visits.

## Notes

Supplement 1.
eMethods

eReferences

Supplement 2.
Data Sharing Statement
