# Supplementary material for: Telemedicine for Initiation of Alcohol Use Disorder Medications
Source: JAMA Netw Open. 2024 Sep 4;7(9):e2431594. doi: 10.1001/jamanetworkopen.2024.31594 (PMC11375471; doi:10.1001/jamanetworkopen.2024.31594)
Supplement: Supplement 1. — eMethods eReferences [file jamanetwopen-e2431594-s001.pdf]

Supplemental Online Content

Huskamp HA, Uscher-Pines L, Raja P, Normand SLT, Mehrotra A, Busch AB. Telemedicine for initiation of alcohol use disorder medications. *JAMA Netw Open*. 2024;7(9):e2431594. doi:10.1001/jamanetworkopen.2024.31594

eMethods

eReferences

This supplemental material has been provided by the authors to give readers additional information about their work.

© 2024 Huskamp HA et al. *JAMA Network Open*

eMethods

We used data on initiations of medications for alcohol use disorder (MAUD) among adults aged 18-64 from Optum Labs Data Warehouse from January 2019 through September 2023. Members of our study cohort included individuals enrolled in commercial insurance plans and in Medicare Advantage plans (i.e., private health plans that contract with the Medicare program to provide coverage to Medicare beneficiaries). The data, which cover all 50 states, have been previously used to describe U.S. health care patterns.[1-6]

We used the following approach to identify MAUD initiations. This approach has been used previously to identify initiations of behavioral health medications.[6-8] We focused on MAUD listed in the table below.

	**Generic name**	**Brand name**
FDA approved	Disulfiram	Antabuse
	Acamprosate	Campral
	Naltrexone	Revia, Depade, Trexan
	Naltrexone	Vivitrol
Not FDA approved	Topiramate	Topamax, Eprontia, Topiragen
	Topiramate	Trokendi XR
	Topiramate	Qudexy XR

We included naltrexone, acamprosate, and disulfiram because they are FDA approved for alcohol use disorder. We also included topiramate because it was the only non-FDA approved ("off-label") medication that was recommended for the treatment of AUD in the most recent VA guidelines on substance use disorders. As per the VA guidelines (healthquality.va.gov/guidelines/MH/sud/VADoDSUDCPG.pdf), these four medications (naltrexone, topiramate, acamprosate, and disulfiram) are considered first-line pharmacotherapy for AUD. We included fills for injectable naltrexone, even though they are not eligible for telemedicine initiation, because our goal is to make statements about the role of telemedicine in prescribing across the entire category of medications that are FDA approved for AUD or appear in treatment guidelines as first-line treatments.

Analyses were conducted using SAS for Windows Version 9.4.

Identifying MAUD initiation

A MAUD initiation was defined as the first MAUD prescription fill during the period (hereafter the "index fill") with no MAUD fills in the previous 90 days. The index fill date was considered the initiation date. We focused on the first MAUD initiation to identify new MAUD use during the period.

Algorithm of Medication Initiation via Telemedicine

To determine whether a MAUD initiation was conducted via telemedicine vs. in-person, we first link the index fill with outpatient visits occurring 7 days prior to or up to 3 days after the index fill. We defined an outpatient visit as claims with a visit for any one of the following outpatient CPT and HCPCS codes: 90791-90792, 90832-90840, 90845-90849, 90853, 90857, 90865, 90867-90871, 90875-90876, 90880, 90900-90902, 90904, 90906, 90908, 90910, 97003, 97004, 98960-98962, 99058, 99078, 99201-99205, 99211-99215, 99241-99245, 99341-99345,

© 2024 Huskamp HA et al. *JAMA Network Open*

99347-99350, 99382-99387, 99392-99397, 99401-99404, 99408, 99409, 99411, 99412, 99420, 99441-99443, 99843, 99490, 99495, 99496, 99510, 0359T-0374T, G0155, G0175, G0351, G0396, G0397, G0438, G0439, G0442, G0443, G0463, G0466-G0470, G0505, G0507, G0513-G0515, G2025, G2067-G2080, G2086-G2088, H0001-H0007, H0014, H016, H0022, H0023, H0028, H0029, H0031, H0034, H0036-H0050, H1011, H2000, H2001, H2010-H2033, H2037, H5010, H5020, H5025, H5030, H5220, H5230, H5240, H5299, M0064, S9454, S9482, S9484, S9485, T1006, T1007, T1011, T1012, T1015-T1018, T1023-T1027, T1040, T1041, T2010-T2015, T2018-T2024, T2036, T2037, Z0001, Z0002

Claims for visits delivered via telemedicine have modifier codes (GT, GQ, 95) or were claims for visits with HCPCS codes specific to telemedicine and potentially applicable to AUD treatment (99441–99443, 98966–98968, G2025).

To identify the outpatient visit associated with the index MAUD treatment, we first looked for outpatient visits with the same provider identifier on the outpatient visit claim as on the index pharmacy claim. If the enrollee had a single outpatient visit with the same provider identifier during that -7/+3 day window around the index fill date (i.e., the 7 days before the index date, the index date itself, and the 2 days after the index date), that visit was considered the initiation visit.

If the enrollee had more than one outpatient visit with the same provider identifier during that window, the initiation visit is considered to be an in-person initiation if any of those visits were conducted in-person; if all of those visits were via telemedicine, it was considered to be a telemedicine initiation.

For index fills with no outpatient visits during the -7/+3 day window with the same provider identifier as the pharmacy claim, we looked for outpatient visits in that window with an AUD diagnosis code in the primary or secondary diagnosis field. If the enrollee had a single visit during this window that met this criterion, that visit was considered the initiation visit. If the enrollee had more than one outpatient visit during that window that met this criterion, the initiation was considered to be an in-person initiation if any of those visits were conducted in-person; if all of those visits were via telemedicine, it was considered to be a telemedicine initiation.

We used the following diagnosis codes to identify acute or chronic alcohol-related medical conditions in the 180 days before and including the MAUD index fill date.

**ICD10 Code**	**100% Attributable Alcohol Related Chronic Conditions**
G62.1	Alcohol polyneuropathy
G31.2	Degeneration of nervous system due to alcohol
G72.1	Alcoholic myopathy
I42.6	Alcohol cardiomyopathy
K29.2	Alcohol gastritis
K70.0-K70.4, K70.9	Alcoholic liver disease
K85.2	Alcohol induced acute pancreatitis
K86.0	Alcohol induced chronic pancreatitis

Source: Classification of Diseases (ICD) Codes and Alcohol-Attributable Fraction (AAF) Sources. https://www.cdc.gov/alcohol/ardi/alcohol-related-icd-codes.html

To identify co-occurring mental illness, we used the following diagnosis codes in the 180 days before and including the MAUD index fill date: ICD-10 F20-F69, F90-F99. To identify co-occurring SUD other than AUD, we used these codes: F11-F16, F18, F19.

Variables included in logistic regression models

Logistic regression models included age category; documented sex; Census region; rural residence; quartiles for median household income in enrollee's county of residence; indicators for whether the enrollee had any claims with a non-AUD SUD diagnosis, with a mental health diagnosis, or with an acute or chronic alcohol-related medical condition diagnosis; an indicator for clinician specialty (psychiatrist or other type of prescriber, relative to a primary care physician (PCP)); and month (where March 2020, the start of the pandemic, is coded as 0) and month squared variables to capture time trends.

© 2024 Huskamp HA et al. *JAMA Network Open*

eReferences

1. Huskamp HA, Busch AB, Uscher-Pines L, Barnett ML, Riedel L, Mehrotra A. Treatment of Opioid Use Disorder Among Commercially Insured Patients in the Context of the COVID-19 Pandemic. JAMA. 2020 Dec 15;324(23):2440-2442. doi: 10.1001/jama.2020.21512. Erratum in: JAMA. 2021 May 11;325(18):1905. PMID: 33320214; PMCID: PMC7739126.

2. Patel SY, Mehrotra A, Huskamp HA, Uscher-Pines L, Ganguli I, Barnett ML. Trends in Outpatient Care Delivery and Telemedicine During the COVID-19 Pandemic in the US. JAMA Intern Med. 2021 Mar 1;181(3):388-391. doi: 10.1001/jamainternmed.2020.5928. PMID: 33196765; PMCID: PMC7670397.

3. Patel SY, McCoy RG, Barnett ML, et al. Diabetes Care and Glycemic Control During the COVID-19 Pandemic in the United States. JAMA Intern Med. Oct 1 2021;181(10):1412-1414. doi:10.1001/jamainternmed.2021.3047

4. Ray KN, Wittman SR, Yabes JG, et al. Telemedicine Visits to Children During the Pandemic: Practice-Based Telemedicine Versus Telemedicine-Only Providers. Acad Pediatr. Mar 2023;23(2):265-270. doi:10.1016/j.acap.2022.05.010

5. Hailu R, Mehrotra A, Huskamp HA, Busch AB, Barnett ML. Telemedicine Use and Quality of Opioid Use Disorder Treatment in the US During the COVID-19 Pandemic. JAMA Netw Open. 2023 Jan 3;6(1):e2252381. doi: 10.1001/jamanetworkopen.2022.52381. PMID: 36692880; PMCID: PMC10038015.

6. Huskamp HA, Uscher-Pines L, Raja P, Normand ST, Mehrotra A, Busch AB. Trends in Use of Telemedicine for Stimulant Initiation Among Children and Adults. Psychiatr Serv. 2024 Jan 19:appips20230421. doi: 10.1176/appi.ps.20230421. Epub ahead of print. PMID: 38239181.

7. Patel SY, Ortiz EG, Barsky BA, Huskamp HA, Busch AB, Mehrotra A. Patient and Clinician Characteristics Associated with Use of Telemedicine for Buprenorphine Induction Among Medicare Beneficiaries. J Gen Intern Med. 2022 Nov;37(14):3758-3761. doi: 10.1007/s11606-022-07633-y. Epub 2022 Apr 29. PMID: 35488099; PMCID: PMC9054110.

8. Barsky BA, Busch AB, Patel SY, Mehrotra A, Huskamp HA. Use of Telemedicine for Buprenorphine Inductions in Patients With Commercial Insurance or Medicare Advantage. JAMA Netw Open. 2022 Jan 4;5(1):e2142531. doi: 10.1001/jamanetworkopen.2021.42531. PMID: 34989798; PMCID: PMC8739765.
