# Supplementary material for: Telemedicine for Initiation of Alcohol Use Disorder Medications
Source: JAMA Netw Open. 2024 Sep 4;7(9):e2431594. doi: 10.1001/jamanetworkopen.2024.31594 (PMC11375471; doi:10.1001/jamanetworkopen.2024.31594)
Supplement: Supplement 2. — Data Sharing Statement [file jamanetwopen-e2431594-s002.pdf]

Data Sharing Statement

Huskamp. Telemedicine for Initiation of Alcohol Use Disorder Medications. *JAMA Netw Open*. Published September 04, 2024. doi:10.1001/jamanetworkopen.2024.31594

Data
Data available: No

Additional Information
Explanation for why data not available: Our data use agreement with Optum Labs does not allow us to make the data available to others. We do provide a detailed supplement with coding and algorithms used so others can replicate our work.
